# Biomimetic Mineralization of Iron-Fumarate Nanoparticles for Protective Encapsulation and Intracellular Delivery of Proteins

**DOI:** 10.1021/acs.chemmater.2c01736

**Published:** 2022-10-03

**Authors:** Negar Mirzazadeh Dizaji, Yi Lin, Thomas Bein, Ernst Wagner, Stefan Wuttke, Ulrich Lächelt, Hanna Engelke

**Affiliations:** † Faculty for Chemistry and Pharmacy, Ludwig-Maximilians-Universität München, Butenandtstr. 5-13, 81377 Munich, Germany; ‡ Center for NanoScience, Ludwig-Maximilians-Universität München, Schellingstr. 4, 80799 Munich, Germany; § Basque Center for Materials (BCMaterials), UPV/EHU Science Park, 48940 Leioa, Spain; ∥ Ikerbasque, Basque Foundation for Science, 48009 Bilbao, Spain; ⊥ Department of Pharmaceutical Sciences, University of Vienna, Josef-Holaubek-Platz 2, 1090 Vienna, Austria; # Department of Pharmaceutical Chemistry, Institute of Pharmaceutical Sciences, University of Graz, Humboldtstr. 46, 8010 Graz, Austria

## Abstract

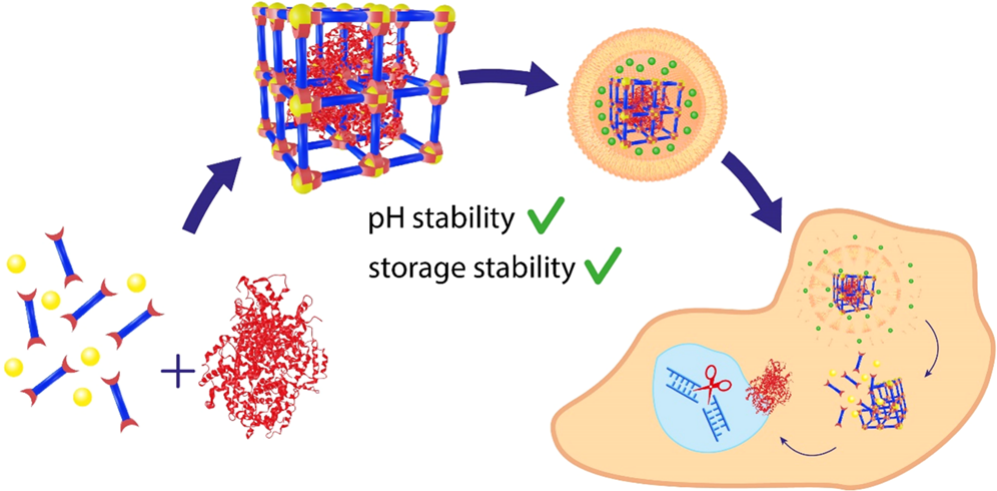

Biomimetic mineralization of proteins and nucleic acids into hybrid metal−organic nanoparticles allows for protection and cellular delivery of these sensitive and generally membrane-impermeable biomolecules. Although the concept is not necessarily restricted to zeolitic imidazolate frameworks (ZIFs), so far reports about intracellular delivery of functional proteins have focused on ZIF structures. Here, we present a green room-temperature synthesis of amorphous iron-fumarate nanoparticles under mildly acidic conditions in water to encapsulate bovine serum albumin (BSA), horseradish peroxidase (HRP), green fluorescent protein (GFP), and Cas9/sgRNA ribonucleoproteins (RNPs). The synthesis conditions preserve the activity of enzymatic model proteins and the resulting nanoparticles deliver functional HRP and Cas9 RNPs into cells. Incorporation into the iron-fumarate nanoparticles preserves and protects the activity of RNPs composed of the acid-sensitive Cas9 protein and hydrolytically labile RNA even during exposure to pH 3.5 and storage for 2 months at 4 °C, which are conditions that strongly impair the functionality of unprotected RNPs. Thus, the biomimetic mineralization into iron-fumarate nanoparticles presents a versatile platform for the delivery of biomolecules and protects them from degradation during storage under challenging conditions.

## Introduction

Biomacromolecules such as RNA and proteins are of great importance for a wide range of applications in the life sciences. The utilization and the exploitation of their potential, however, face several challenges, particularly with respect to their stability and delivery into cells. Unlike many small molecules, they are not readily internalized by cells and most of them have to be delivered, also in in vitro settings.^1^ Thus, even for in vitro applications, suitable carrier systems have to be designed. Furthermore, the large size of many biomacromolecules impedes loading into the small pores of most common porous nanomaterials. Additionally, most of the employed biomolecules are fragile and need to be protected from harsh conditions as well as hydrolytic, enzymatic, or other degradation mechanisms. The accommodation of biomolecules in larger pores of inorganic nanoparticles or in polymeric nanoparticles has been achieved.^2,3^ Similarly, delivery of various proteins and nucleic acids has been achieved via lipid^4^ or polymer nanoparticles,^5^ large-pore inorganic^6,7^ or hybrid nanoparticles,^8,9^ as well as by addition of cell-penetrating peptides^10,11^ and other modifications that facilitate intracellular delivery. However, protection of the sensitive cargo from degradation still remains a major challenge.

A seminal work on the biomimetic mineralization of proteins into metal−organic framework (MOF) nanoparticles has revealed a solution for protecting biomolecules from very harsh conditions.^12,13^ In this approach, MOFs are synthesized in presence of the protein, incorporating it into the structure and releasing it upon MOF degradation.^12,14^ Incorporation into the MOF structure protects the proteins from heat and other harsh conditions.^12^ This technique of MOF-based biomimetic mineralization has been successfully applied to generate nanobiocomposites, which deliver a variety of proteins into cells, such as antibodies,^15^ caspases,^16^ and Cas9.^14,17^ These applications were mainly based on the zinc imidazolate framework (ZIF) materials ZIF-8 and ZIF-90, which consist of Zn and imidazole derivatives.^18^ A reason for selecting these ZIF structures is that they can be synthesized under aqueous conditions, at room temperature, and physiological pH. The imidazoles within the ZIF structures get protonated at slightly acidic pH,^19^ which is favorable for disassembly and cargo release within endo- and lysosomes.^14^ However, the characteristic of being readily degradable can cause issues for storage. Furthermore, Zn ions are important signaling messengers and can be very toxic to cells.^20^ Therefore, nanoparticles alternative to ZIF-8 and ZIF-90 are needed for biomimetic mineralization under conditions that preserve proteins and their function.

**Figure 1:**
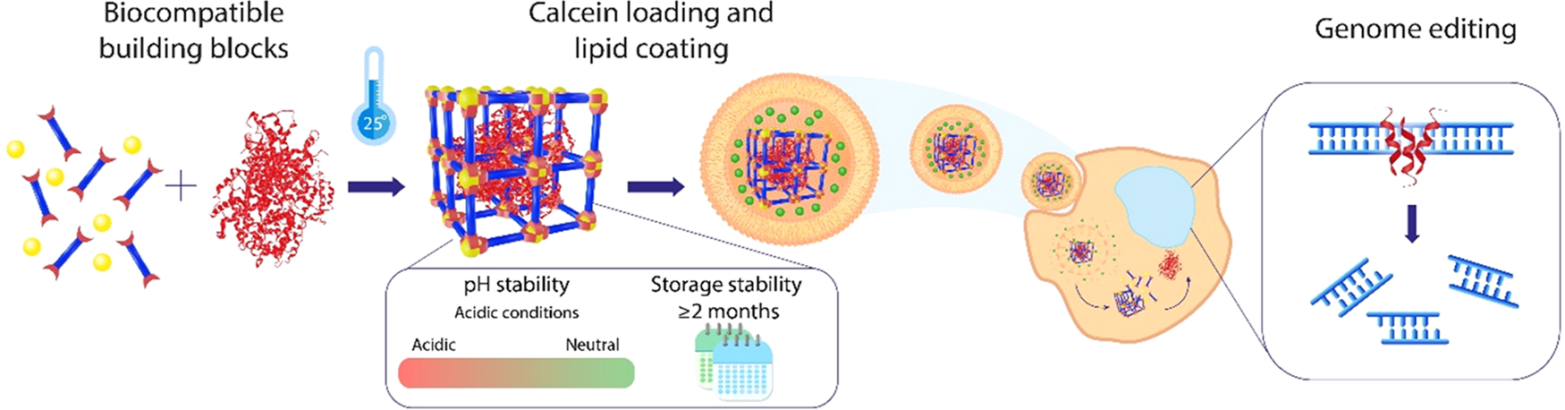
Overview of synthesis and protective properties of Fe-fum nanoparticles, as well as their use for intracellular delivery of functional proteins, such as Cas9/sgRNA RNPs for genome editing.

Iron-fumarate nanoparticles, such as MIL-88A, have shown great promise for drug delivery.^21,22^ They can be imaged via MRI^23,24^ and have been used to deliver small molecules to cells.^21,25^ Both building blocks—fumaric acid and iron—are naturally present in the human body and therefore relatively well tolerated.^26,27^ A study on cytotoxicity of MOF nanoparticles based on Fe, Zn, and Zr showed that Fe-based MOFs were less toxic to HeLa and J774 cells than Zn and Zr MOFs.^28^ Iron-fumarate nanoparticles can be synthesized via biomimetic mineralization to include BSA.^12^ However, so far, the synthesis procedure was performed at acidic pH 2.5 that is not tolerated by many proteins. For example, Cas9, which receives much attention as a highly flexible gene editing tool, has been reported to irreversibly loose its bioactivity upon exposure to acidic pH < 4.^29^ Here, we introduce a biomimetic mineralization of proteins with iron-fumarate nanoparticles at pH 4.8, which preserves and shields pH-sensitive protein structures. We show that iron-fumarate nanoparticles can be used as an alternative to ZIF nanoparticles for delivery of proteins, such as Cas9. Importantly, the iron-fumarate platform preserves protein functionality during synthesis, delivery, release, and even under harsh conditions such as storage at acidic pH or in ethanol (Figure 1).

## Results

### Synthesis and Characterization of Biomimetically Mineralized Iron-Fumarate Nanoparticles

**Figure 2:**
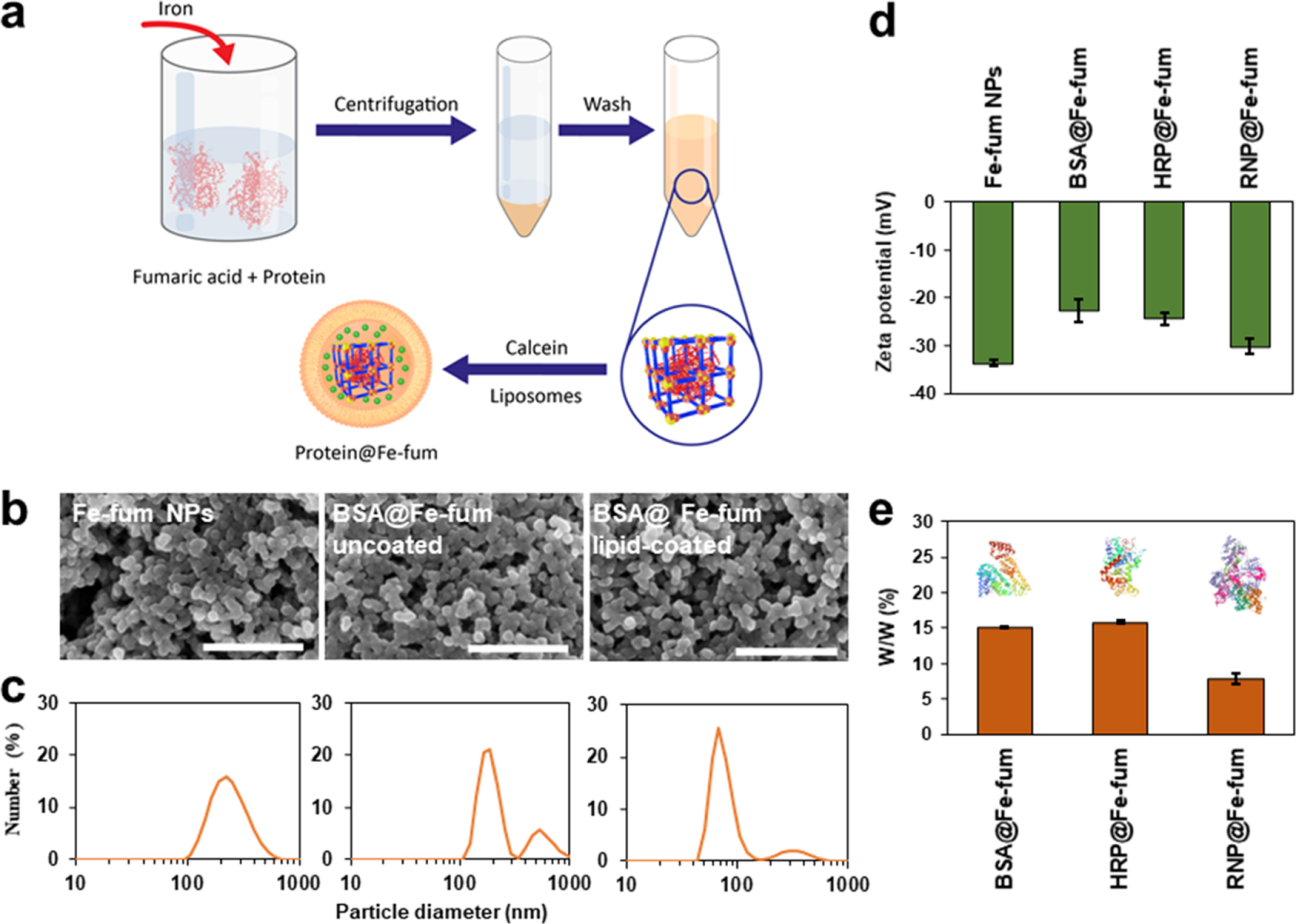
Preparation and characterization of Fe-fum NPs and protein@Fe-fum. (a) Schematic of biomineralization of Fe-fum NPs. (b) SEM images (scale bar: 400 nm) and (c) size distribution obtained via dynamic light scattering (DLS) measurements of Fe-fum NPs, BSA@Fe-fum without lipid coating, and lipid-coated BSA@Fe-fum, respectively. (d) ζ potential of Fe-fum NPs and protein@Fe-fum dispersed in water; proteins include BSA, HRP, and Cas9/sgRNA RNP. (e) Quantification of encapsulated proteins (BSA, HRP, and Cas9/sgRNA RNP) assessed by disintegration of uncoated NPs and a subsequent BCA assay.

In previous protocols, spherical iron-fumarate nanoparticles were synthesized via room-temperature precipitation from water-based solutions of fumaric acid and iron chloride.^21,25,26^ Due to the fumaric acid, this occurs at a pH of 2.5. To accommodate proteins and preserve their structure and function, we developed a synthesis protocol at less acidic pH. Increasing the pH decreases protonation of fumaric acid and thus its reactivity with iron and subsequent nanoparticle formation. Therefore, the pH had to be balanced to preserve protein function and at the same time allow for nanoparticle formation, yielding pH 4.8 as best compromise. To compensate for the reduced reactivity of fumaric acid, the molar ratio of fumaric acid to iron was increased from 1:1 to 10:1. Briefly, nanoparticle formation was initiated by incubation of proteins with fumaric acid at pH 4.8, followed by addition of iron chloride (Figure 2a). Subsequently, the resulting iron-fumarate nanoparticles (Fe-fum NPs) were washed with ethanol. For cell experiments, the particles were loaded with calcein and coated with a lipid layer via a fusion method as described previously.^21^ The coencapsulated calcein enabled visualization by fluorescence microscopy and additionally stabilized the lipid layer.^30^ As observed previously, lipid coating facilitates cellular uptake of iron-fumarate-based nanoparticles and thereby contributes to successful delivery.^21^ To confirm that Fe-fum NPs can serve as a versatile platform for protective encapsulation and intracellular delivery of proteins, different proteins were encapsulated: bovine serum albumin (BSA), horseradish peroxidase (HRP), green fluorescent protein (GFP), and Cas9/sgRNA ribonucleoproteins (RNPs).

A detailed materials characterization was carried out with BSA biomimetically mineralized into Fe-fum NPs (BSA@Fe-fum). Scanning electron microscopy (SEM) reveals a spherical morphology and an average size of 30 nm (Figures 2b and S1). As displayed in the SEM images in Figure 2b, morphology and size of Fe-fum NPs did not change upon incorporation of BSA nor upon lipid coating. Dynamic light scattering (DLS, Figure 2c) of the Fe-fum NPs without incorporated proteins shows a homogeneous size distribution around a hydrodynamic diameter of 220 nm, which is within a suitable range for drug delivery purposes. After incorporation of BSA, the majority of Fe-fum NPs showed a size distribution around 200 nm. However, a second population with larger sizes appeared, indicating a certain degree of aggregation. DLS of lipid-coated Fe-fum NPs resulted in a size distribution of the main fraction of nanoparticles around 70 nm. All lipid-coated Fe-fum NPs exhibited a negative ζ potential. The ζ potential of lipid-coated Fe-fum NPs without protein was −33 mV, which was slightly increased upon incorporation of proteins as displayed in Figure 2d.

X-ray diffraction (XRD, Figure S2) and infrared spectroscopy (IR) data (Figure S3) are consistent with data found in the literature for other iron-fumarate nanoparticles.^26^ Particularly, the amorphous nature revealed by XRD has been described for iron(III) fumarate nanoparticles with similarly spherical shape.^26^ IR was further used to assess the incorporation of HRP and Cas9 RNP into the Fe-fum NPs. In the IR spectra, the peak in the range of 1600–1710 cm^–1^ represents a typical protein signal (corresponding to the amide I band, mainly from C=O stretching vibrations).^31^ It is present in the spectra of Fe-fum NPs incorporating HRP and Cas9 RNP, and it is absent in the spectrum of Fe-fum NPs without incorporated proteins. This confirms that HRP and Cas9 RNP were successfully incorporated in the Fe-fum NPs (SI, Figure S3).

Protein encapsulation was further quantified with a BCA (bicinchoninic acid) assay of Fe-fum NPs degraded with citrate buffer. The protein loading efficiency was calculated as the percentage of protein incorporated in Fe-fum NPs relative to the total initial protein provided during synthesis, and the protein loading capacity was quantified as the weight percentage of the incorporated protein per mg Fe-fum NPs. For BSA and HRP, loading efficiencies of 84 and 86%, respectively, and loading capacities of 15 and 15.7%, respectively, were obtained. The same initial concentration of Cas9 RNP yielded a lower loading efficiency of 38% and lower loading capacity of 7.8% (Figure 2e). The reduced loading efficiencies and capacities for Cas9 may be attributed to its physical and chemical characteristics, which differ from BSA and HRP. Most likely, it is a result of its much larger size (160 kDa) compared to BSA (69 kDa) and HRP (44 kDa), which might lead to steric effects reducing the packing efficiency. Nevertheless, compared to Cas9 loading into ZIF nanoparticles reported in the literature^14^ (1.2%), the loading capacity of 7.8% that we achieve with Fe-fum NPs is considerably higher.

To assess whether Fe-fum NP synthesis preserves protein function, we initially used GFP fluorescence as a marker. While GFP fluorescence was strongly reduced during synthesis at pH 2.5, the synthesis at pH 4.8 described here preserved GFP fluorescence (Figure S4).

### Intracellular Delivery of BSA

**Figure 3:**
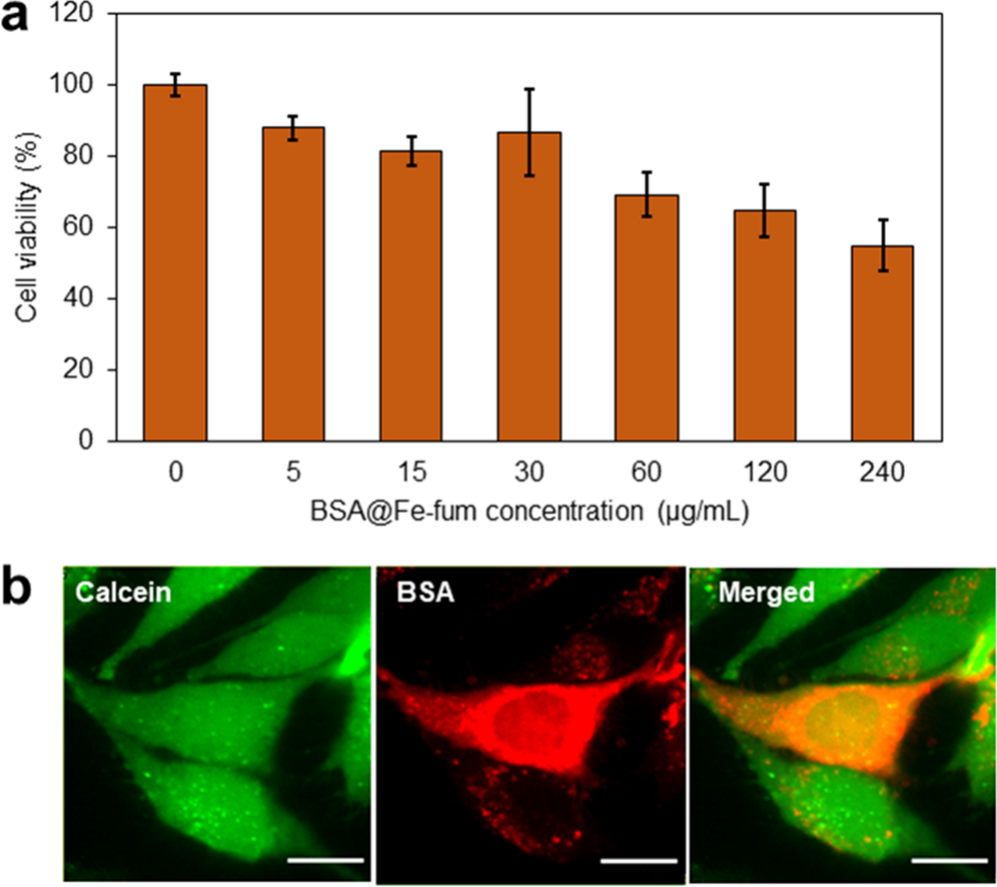
Cell viability and release of BSA@Fe-fum. (a) Viability of HeLa cells treated with lipid-coated, calcein-loaded BSA@Fe-fum for 48 h measured with an MTT assay. (b) Confocal images of HeLa cells incubated with lipid-coated, calcein-loaded Atto633-BSA@Fe-fum for 3 days. Green: calcein, red: Atto633-labeled BSA. Scale bar:10 μm.

Next, we studied the potential of the Fe-fum NPs to deliver proteins into cells using three different proteins of increasing complexity. First, we used BSA fluorescently labeled with Atto633 as a model protein to assess intracellular release and effects on cell viability. The metabolic activity of HeLa cells was determined by MTT assay after 48 h incubation with various amounts of lipid-coated, calcein-loaded BSA@Fe-fum (0–240 μg/mL). Up to a concentration of 30 μg/mL BSA@Fe-fum, no obvious reduction of cell viability could be observed compared to untreated controls. Even at the highest dose of 240 μg/mL, the effect on cell viability was less than 50% (Figure 3a). We then used fluorescence microscopy to monitor the intracellular localization and release of the fluorescent cargos calcein and Atto633-BSA. To observe significant intracellular release, we applied a brief osmotic shock by exposing the cells to 1 M glucose for 6 mins followed by replacement with fresh medium. While the glucose shock may be a limitation for therapeutic applications, it also provides the advantage of temporal control of endosomal release and intracellular activity for applications in biotechnology or research on cell biology. The homogeneous distribution of both calcein and Atto633-BSA in the cells after glucose shock suggests intracellular degradation of the Fe-fum NPs and release of the contained fluorescent cargos (Figure 3b).

### Intracellular Delivery and Activity of HRP

**Figure 4:**
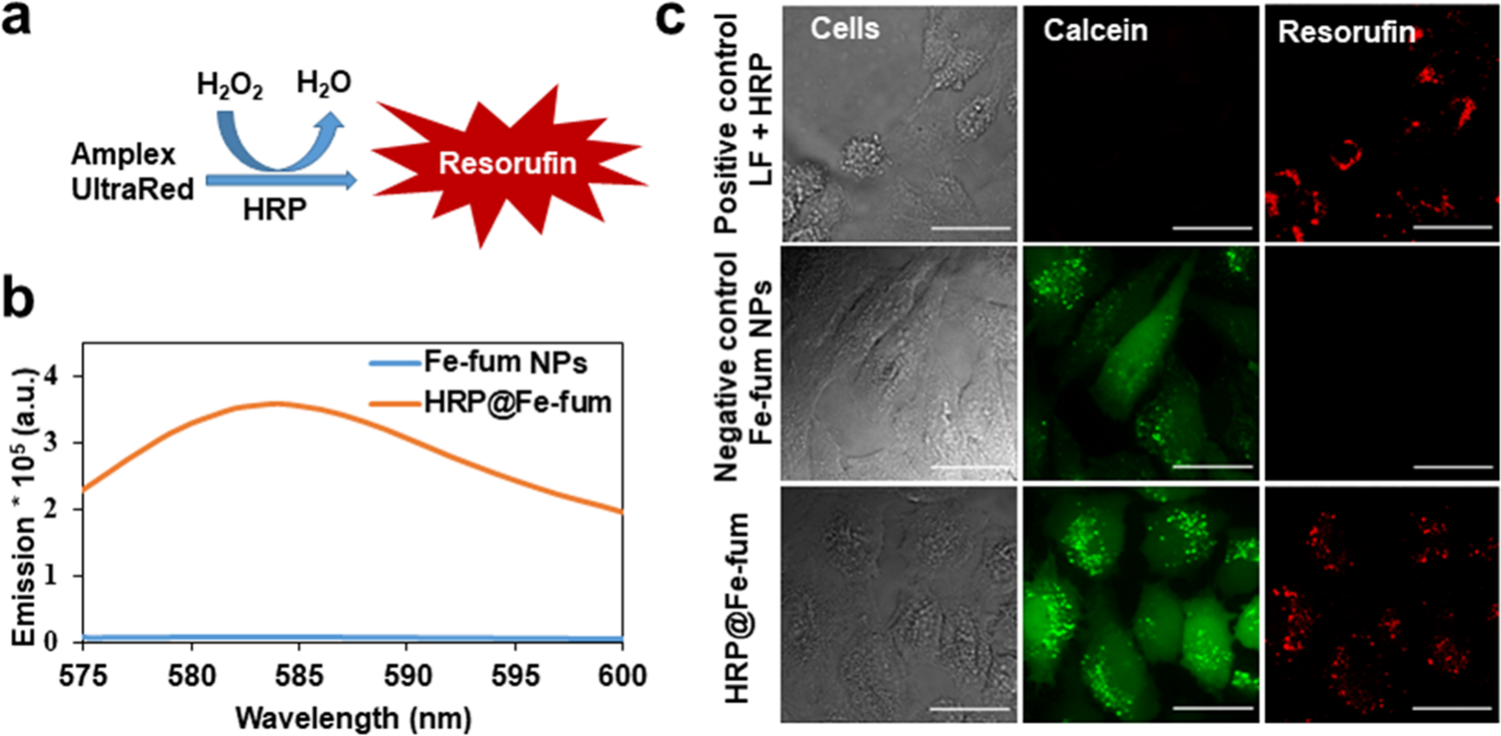
Intracellular activity of HRP delivered by HRP@Fe-fum. (a) Catalytic reaction of HRP with Amplex UltraRed resulting in the formation of fluorescent resorufin. (b) Fluorescence emission after exposure of Amplex UltraRed to disintegrated Fe-fum NPs and HRP@Fe-fum shows activity of HRP after release from HRP@Fe-fum (orange), while controls without HRP do not show activity (blue). (c) Confocal microscopy images of HeLa cells after incubation with lipofectamine (LF) and HRP as a positive control for HRP-induced Amplex UltraRed fluorescence signal; lipid-coated, calcein-loaded Fe-fum NPs without HRP as a negative control; and lipid-coated, calcein-loaded HRP@Fe-fum showing that HRP@Fe-fum NPs release calcein (green channel) and deliver active HRP inducing formation of fluorescent resorufin (red channel). Scale bar: 20 μm.

To observe protein function after intracellular delivery, we investigated the delivery of horseradish peroxidase (HRP). HRP is a widely used enzyme that catalyzes the oxidation of a variety of organic substrates by means of hydrogen peroxide. To assess the functionality of HRP, we therefore used Amplex UltraRed as a substrate, which is a nonfluorescent molecule that is converted to fluorescent resorufin upon HRP-catalyzed oxidation by hydrogen peroxide (Figure 4a).^32,33^ Before cell experiments, we tested the activity of HRP after incorporation into the Fe-fum NPs. To this end, Fe-fum NPs with biomimetically incorporated HRP (HRP@Fe-fum) were disintegrated by incubation in citrate buffer. Subsequently, the disintegrated HRP@Fe-fum were incubated in a solution of Amplex UltraRed and hydrogen peroxide. As shown in Figure 4b, fluorescence emission was detected in the disintegrated HRP@Fe-fum, but not in controls of Fe-fum NPs without HRP. Comparing the enzyme kinetics between HRP released from degraded HRP@Fe-fum and free HRP added to degraded Fe-fum NPs, we found that the Michaelis–Menten constant *K*_M_, i.e., the substrate–enzyme binding rates, is comparable within errors (182 ± 73 μM for free HRP and 127 ± 39 μM for HRP from HRP@Fe-fum). These values are similar to *K*_M_ values for HRP reported in the literature.^34^ However, the catalytic rate of the enzyme *k*_cat_ is reduced in HRP released from degraded HRP@Fe-fum to about 2.5% of the value of free HRP. This is in accordance with reports in the literature on interactions of carboxylic acids with the heme group of the active site of horseradish peroxidase, which lead to a reduction of the enzyme activity rate to a similar extent as we observe for HRP released from Fe-fum NPs.^35^ Thus, the activity of HRP was reduced but still clearly detectable after synthesis, encapsulation, and release. Therefore, we next studied its enzymatic activity after intracellular delivery using the same assay of Amplex UltraRed oxidation to fluorescent resorufin. HeLa cells were incubated with lipid-coated, calcein-loaded HRP@Fe-fum and treated with hydrogen peroxide and Amplex UltraRed. The widespread green fluorescent signal in Figure 4c suggests an efficient release of calcein after glucose shock. Importantly, we observed a red fluorescent signal resulting from resorufin formation in cells incubated with HRP@Fe-fum, but not in control cells treated with Fe-fum NPs without HRP. The resorufin signal was also observed in positive controls employing HRP transfected with lipofectamine. Cell morphology changed in cells incubated with HRP@Fe-fum and in positive controls, but not in negative controls without HRP. Lipid oxidation is known to induce such morphology and can ultimately lead to cell death. The morphology changes may thus result from HRP-catalyzed oxidation of lipids or other important cell constituents. All in all, the observed fluorescence signal and cell morphology suggest active HRP to be successfully delivered into cells with HRP@Fe-fum.

### Intracellular Delivery, Activity, and Preservation of Cas9/sgRNA RNPs

**Figure 5:**
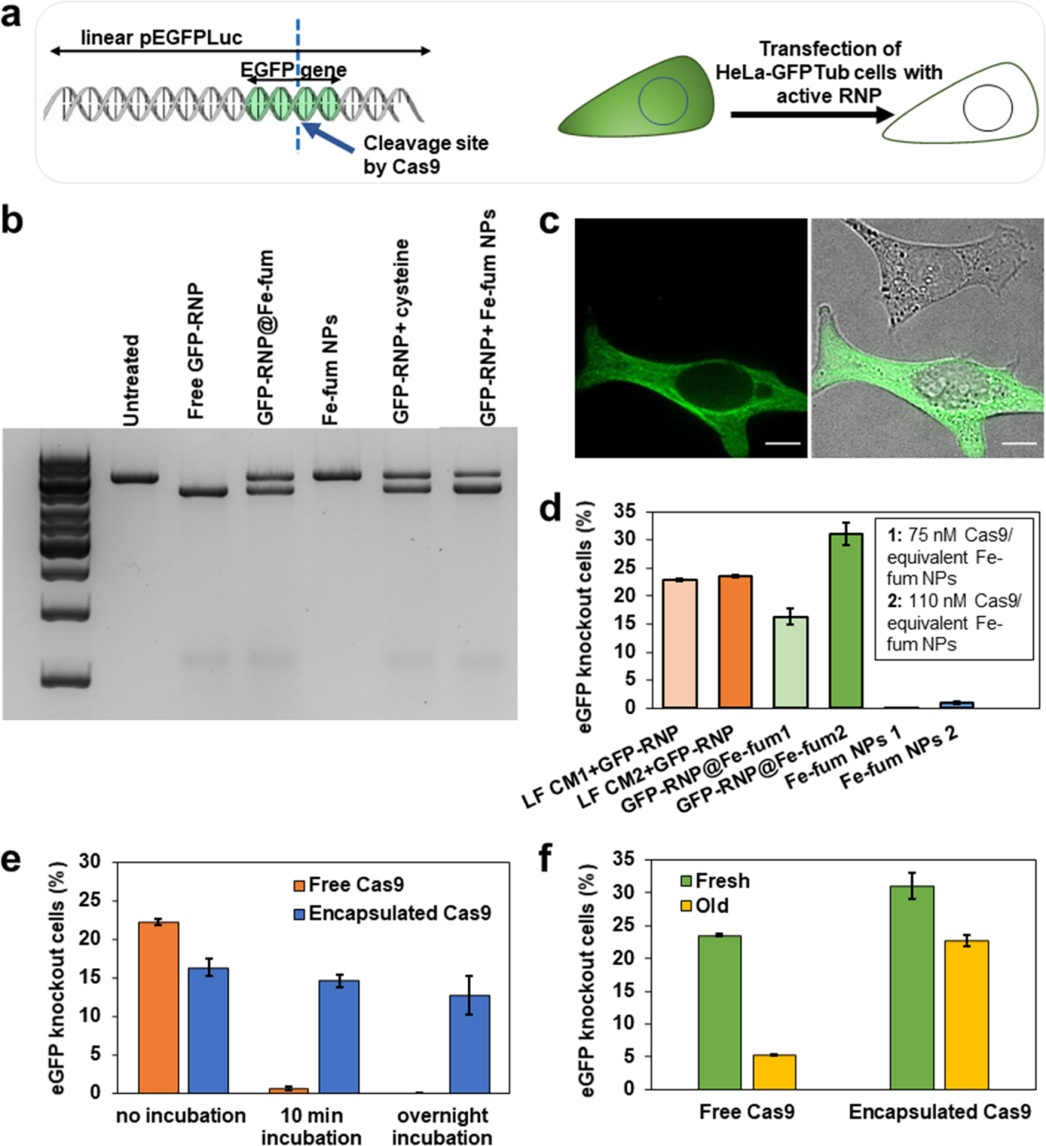
In vitro and intracellular activity of Cas9 RNPs. (a) Schematic of the cleavage of the EGFP gene and cellular knockout caused by Cas9/sgGFP RNPs. (b) Agarose gel electrophoresis of cleavage assay confirming RNP activity after RNP@Fe-fum synthesis. The samples were an untreated control, free RNP, degraded RNP@Fe-fum, degraded Fe-fum NPs without RNP, RNP added to degraded Fe-fum NPs after NP degradation, and RNP exposed to 80 mM cysteine (concentration used for Fe-fum NP degradation). (c) Microscopy images of a cell with GFP-tubulin expression (bottom) and knocked out GFP (top). The left image represents the GFP channel, and the right image the overlay with brightfield. Scale bar: 10 μm (d) Knockout efficiency of lipid-coated, calcein-loaded RNP@Fe-fum corresponding to two different concentrations of RNP: 75 nM (RNP@Fe-fum 1) and 110 nM Cas9 RNP (RNP@Fe-fum 2). The corresponding amounts of Fe-fum NPs without RNP served as negative controls. As positive controls, cells were transfected with the same concentrations of Cas9/sgGFP RNP using Lipofectamine CRISPRMAX (LF CM). LF CM1 and LF CM2 contain 75 nM and 110 nM Cas9, respectively. (e) The impact of acidic conditions and (f) a 2-month storage at 4 °C on the knockout efficiency of encapsulated and free RNP. Free RNP was delivered into the cells via lipofectamine. EGFP knockout efficiencies were determined by flow cytometry. Data are presented as mean ± SD (*n* = 3).

CRISPR/Cas technology is emerging as a key tool for applications in therapy and biotechnology.^36,37^ For this technology to work, ribonucleoprotein (RNP) complexes of Cas9 protein and single-guide RNA (sgRNA) have to act in concert inside cells.^38^ Within this complex, Cas9 is a programmable endonuclease, which cleaves DNA at a target site as guided by the sgRNA. Cas9/sgRNA RNPs,^39^ however, are very sensitive to pH and hydrolysis, complicating storage and thus their application. Therefore, we next tested the potential of Fe-fum NPs to protect and deliver active Cas9/sgRNA RNPs into cells. As for the investigations with HRP, we assessed the preservation of RNP activity during Fe-fum NP synthesis, encapsulation, and release before cell experiments. To this end, an in vitro cleavage assay was carried out, which allows for the determination of the sequence-specific nuclease activity of Cas9/sgRNA RNPs (Figure 5a). Briefly, in this assay, a linearized plasmid containing the EGFP gene (pEGFPLuc) was incubated with the RNP comprised of Cas9 and an EGFP-specific sgRNA (sgGFP). In case of active RNPs, the endonuclease Cas9 cleaves the plasmid into two fragments (SI, Figure S5a), which can be separated and detected by gel electrophoresis. The relative band intensities of the linearized plasmid versus the resulting fragments can be used as a measure of RNP activity. As shown in Figure S5b, incubation in fumaric acid at pH values down to pH 4.8—a pH that is not tolerated by Cas9 RNPs in absence of fumaric acid—did not reduce RNP activity suggesting that fumaric acid protected the RNP complex from pH-induced deactivation. Incubation of Cas9/sgRNA RNPs with degraded Fe-fum NPs slightly reduced the activity of RNPs, but nevertheless showed very high activity (Figure S6). Also, the cleavage assay of degraded Fe-fum NPs containing biomimetically incorporated Cas9/sgRNA RNPs (RNP@Fe-fum) revealed cleavage of the substrate (Figure 5b); however, cleavage efficiency was reduced compared to free RNP controls. This reduction in cleavage efficiency may result from the conditions used for degradation of the Fe-fum NPs. In particular, the cysteine used for degradation of Fe-fum NPs can reduce RNP activity, as shown by the control of free RNPs directly incubated with cysteine (Figure S6, for details on degradation by cysteine see Figures S7 and S8). Even though we cannot fully exclude that the observed reduction in activity occurs during synthesis or during degradation of the loaded RNP@Fe-fum in the cell, the observed activity encourages further experiments on intracellular delivery. Therefore, we next analyzed the efficiency of the biomimetically mineralized RNP@Fe-fum to mediate gene knockout in cells. To this end, we incubated HeLa cells expressing GFP-tubulin (Hela GFPtub) with lipid-coated, calcein-loaded RNP@Fe-fum that contained the same sgRNA targeting the GFP coding region used in the in vitro cleavage assay. In case of successful intracellular delivery, the cleavage of the GFP gene leads to gene knockout and loss of fluorescence. This is depicted in Figure 5c, which shows a cell with GFP-tubulin and a cell without fluorescence upon GFP knockout. The knockout efficiency after cellular treatments was quantified by flow cytometry. To exclude artifacts due to codelivered calcein, which has a fluorescence spectrum overlapping with GFP, the cells were passaged twice and regrown for two days after each passage (Figure S9). After treatment with lipid-coated, calcein-loaded RNP@Fe-fum at concentrations corresponding to 75 and 110 nM Cas9 RNP, knockout efficiencies amounted to 16 and 30%, respectively (Figure 5d). Lipofectamine CRISPRMAX (LF CM), which was used as a commercially available benchmark reagent, yielded knockout efficiencies of 22 and 23% at the same concentrations of 75 and 110 nM Cas9 RNP, respectively. Hence, RNP@Fe-fum mediated higher knockout levels at 110 nM RNP compared to LF CM. In contrast, negative controls of Fe-fum NPs without RNP and Fe-fum or LF with a control sgRNA, without a target sequence in the genome, did not show GFP knockout (Figures S10–S12). With these knockout efficiencies, biomimetically mineralized iron-fumarate nanoparticles may represent a good alternative for the delivery of Cas9/sgRNA RNPs into cells.

Next, we studied the ability of biomimetically mineralized Fe-fum NPs to protect Cas9/sgRNA RNPs from degradation under challenging conditions. Importantly, all RNP@Fe-fum are washed and stored in ethanol after synthesis and before lipid coating. Thus, the knockout efficiencies obtained above already show that Fe-fum NPs successfully protect RNPs from a potential negative impact of ethanol. RNPs and many other proteins lose their activity under acidic conditions.^29,40^ Therefore, we assessed the ability of biomimetically mineralized Fe-fum NPs to protect RNPs from acidic pH. The experiments shown above on the protection of RNPs by fumaric acid at pH 4.8, which otherwise inactivates RNPs, already suggest that RNPs might also be protected in the Fe-fum NPs. To further investigate the potential protection from acidic conditions, we exposed lipid-coated, calcein-loaded RNP@Fe-fum to an acidic environment (pH 3.5) for a short-term (10 min) and a long-term (overnight) incubation. The stability of Fe-fum NPs was confirmed by UV/VIS spectrometry (Figure S13), and subsequently, the RNP activity was measured via cellular gene knockout efficiency. While a control of free and unprotected RNP, which was incubated under the same conditions and subsequently transfected with lipofectamine, lost its activity already after 10 min incubation at pH 3.5, RNPs incorporated into Fe-fum NPs retained their activity and achieved similar knockout levels even after overnight incubation at acidic pH (Figure 5e). In addition to the protective properties against acidic conditions, we also evaluated the ability of biomimetically mineralized Fe-fum nanoparticles to facilitate long-term storage. Again, free RNPs stored for 2 months at 4 °C and transfected with lipofectamine lost almost all its cellular knockout efficiency. In contrast, RNP@Fe-fum stored for the same time at 4 °C achieved almost the same knockout levels as the fresh RNP@Fe-fum (Figure 5f). Thus, the biomimetically mineralized Fe-fum NPs provide good protection of incorporated Cas9/sgRNA RNPs and are a promising storage form.

## Conclusions

In conclusion, we have developed a synthesis protocol for biomimetically mineralized iron-fumarate nanoparticles that preserves proteins and their function. The resulting Fe-fum NPs had comparably high loading efficiency of proteins and successfully delivered them into cells preserving protein activity. Delivery of Cas9/sgRNA RNPs showed efficient gene knockout in HeLa cells. While other efficient delivery systems for proteins exist,^5^ generic strategies for flexible encapsulation of different proteins and their protection from degrading conditions are still challenges. The biomineralization approach presented here is a versatile platform as shown by the four different encapsulated model proteins (BSA, GFP, HRP, Cas9/sgRNA RNPs). Furthermore, the biomimetically mineralized Fe-fum NPs provide very good protection of Cas9/sgRNA RNPs against acidic pH and allowed for storage over 2 months at 4 °C. They do not show efficient release without glucose shock, which demands further research for on-board release triggers for therapeutic applications. Yet, the glucose shock allows for temporal control of release in applications in biotechnology and cell biology research. Thus, the Fe-fum NPs present a valuable alternative to existing ZIF or polymer delivery systems, specifically when protein protection or temporal control of release is necessary.

## Experimental Section

### Materials

Chemicals were purchased from Sigma-Aldrich (St. Louis, MO), if not stated otherwise.

### Biomimetic Mineralization of Protein@Fe-fum

Twenty milliliters of a 10 mM solution of fumaric acid in deionized water was prepared, and the pH of the solution was adjusted to 4.8 by adding NaOH. Proteins were added into the solution of fumaric acid in the concentration range of 30–150 μg/mL. The mixture was incubated for 10 min at room temperature and 750 rpm stirring. A separate solution of iron chloride in deionized water (10 mM, 2 mL) was prepared. Then, the iron chloride solution was added to the fumaric acid and protein mixture in 5 steps at 20 s intervals. The resulting protein@Fe-fum were washed three times by centrifugation at 7179 RCF for 20 min and subsequent redispersion in ethanol.

### Production and Purification of Cas9 Protein

Production and purification of Cas9 protein were performed as previously reported.^5^ pET28a/Cas9-Cys was a gift from Hyongbum Kim^41^ (Addgene plasmid # 53261; http://n2t.net/addgene:53261; RRID:Addgene_53261). In brief, the plasmid pET28a/Cas9-Cys was transformed into Rosetta BL21 (DE3) pLysS competent cells (Merck Millipore, Germany). A monoclonal culture of the bacteria was cultivated in LB medium (34 μg/mL chloramphenicol and 50 μg/mL kanamycin) under shaking (250 rpm) at 37 °C until an optical density at 600 nm of 0.7 was reached. Subsequently, 1 mM isopropyl β-d-1-thiogalactopyranoside (IPTG) was added to induce Cas9 protein expression. Bacteria were then harvested and lysed. Purification of Cas9 protein was conducted by nickel chromatography (HisTrap HP column, GE Healthcare, Sweden) using a gradient from the binding buffer (20 mM Trizma base, 0.5 M NaCl, pH 7.4, 20 mM imidazole) to elution buffer (20 mM Trizma base, 0.5 M NaCl, pH 7.4, 0.5 M imidazole). The fractions containing Cas9 were collected and further purified by size exclusion chromatography on an Äkta purifier system using the storage buffer (20 mM HEPES, 200 mM KCl, 10 mM MgCl_2_, and 1 mM DTT) as the mobile phase. The fractions containing Cas9 were combined, and the concentration of Cas9 was measured using a NanoDrop photometer (Thermo Scientific). The Cas9 solution was aliquoted and stored at −80 °C before use.

### Preparing Ribonucleoprotein (RNP) Complexes

Cas9/sgGFP RNP complexes were formed by mixing Cas9 protein with sgGFP (spacer sequence: GACCAGGAUGGGCACCACCC) or control sgRNA (space sequence: GGGTAACCGTGCGGTCGTAC) at a molar ratio of 1:1 at room temperature (RT) for 15 min. The obtained RNP complexes were diluted in HEPES buffer (20 mM, pH 7.4) to a final concentration of 1.5 μg/μL RNP and directly used for in vitro cleavage assay or preparation of nanoparticles.

### Preparing Labeled Proteins (Atto-BSA and Atto-HRP)

BSA or HRP in solution (3 mg/mL) was labeled with ATTO633-NHS ester fluorescence dye (ATTO-TEC, Siegen, Germany) based on the manufacturer’s instruction. In brief, the pH of the protein solution was adjusted to 8.3 with a 0.2 M sodium bicarbonate solution and then incubated with dye at room temperature for 1 h in the dark. The unbound dye was removed with Bio-Spin 6 size exclusion spin columns (Bio-Rad Laboratories).

### Biomimetic Mineralization of RNP@Fe-fum

RNP was mixed with a fumaric acid solution (10 mM, 5 mL, pH 4.8) to yield a concentration of 30 μg/mL RNP. The mixture was incubated at room temperature for 10 min and 750 rpm. To the mixture of fumaric acid and RNP, 500 μL of iron chloride solution (10 mM) was added in 5 steps at 20 s intervals. The resulting RNP@Fe-fum were washed three times via centrifugation and redispersion in ethanol.

### Preparation of the Calcein-Loaded and Liposome-Coated Fe-fum NPs

A 1 mM solution of calcein in deionized water was prepared. One milligram of Fe-fum NPs or protein@Fe-fum was redispersed in 1 mL of calcein solution. The mixture was then incubated overnight and at 700 rpm shaking for loading. In the case of protein@Fe-fum, the incubation was performed at 4 °C. Then, the Fe-fum NPs were centrifuged for 5 min at 14 000 rpm and the supernatant was discarded to collect the Fe-fum NPs for liposome coating. The liposome coating of the Fe-fum NPs was performed via a fusion method reported by Illes et al.^21^ In this approach, first, a liposome coating solution was prepared by extruding a 1 mg/mL PBS solution of DOPC (1,2-dioleoyl-*sn*-glycero-3-phosphocholine, Avanti) through an extruder with a 100 nm pore-sized membrane 11 times. Then, the pellet of the calcein-loaded Fe-fum NPs or calcein-loaded protein@Fe-fum was redispersed in 500 μL of liposome solution, followed by the addition of 500 μL of deionized water and incubation for 2 h. The particles were then centrifuged (5 min at 14 000 rpm) and redispersed in PBS. All cell experiments were performed with lipid-coated, calcein-loaded nanoparticles.

### Dynamic Light Scattering (DLS)

DLS and ζ potential measurements were performed by applying a Zetasizer Nano series (Nano-ZS, Malvern) equipped with a laser with the wavelength λ of 633 nm. DLS measurements were performed at 25 °C and PMMA cuvettes were used. Samples for DLS measurements were prepared by diluting the freshly produced NPs or liposome-coated NPs in ethanol or PBS, respectively. Samples in water were used for the single-point measurements of the ζ potential of NPs using DTS107 cuvettes. For autotitration measurements, the additional Zetasizer titration system (MPT-2) based on diluted NaOH and HCl as titrants was used. Samples for this purpose were prepared by diluting the Fe-fum NPs to the concentration of 0.1 mg/mL in water.

### Scanning Electron Microscopy (SEM)

All SEM micrographs were recorded with a Helios NanoLab G3UC (FEI) operating at 5 kV. For sample preparation, the Fe-fum NP dispersion was dried overnight on a carbon film placed on an aluminum sample holder followed by carbon sputtering before the measurement. For evaluation of the SEM micrographs, the software ImageJ v1.49 was used.

### X-ray Diffraction (XRD)

XRD experiments were performed on dried Fe-fum NPs or Atto-BSA@Fe-fum (before calcein loading). The samples were measured on a STOE diffractometer system STADI P operating in transmission mode. The setup is using Cu Kα1 radiation with a wavelength λ of 0.15418 nm.

### Infrared Spectroscopy (IR)

Infrared spectra of dried sample powder were recorded on a Thermo Scientific Nicolet iN10 IR microscope in reflection–absorption mode with a liquid N2-cooled MCT-A detector.

### UV Absorbance

Fe-fum NP stability in acidic conditions was studied by UV–vis measurements performed with the Thermo Scientific NanoDrop 2000c spectrometer. Before measurements, Fe-fum NPs were treated with HCl to reach the pH 3.5, and after 10 min, the pH was neutralized by adding NaOH.

### Bicinchoninic Acid Assay (BCA Assay)

To estimate the protein loading efficiency in the protein@Fe-fum, the encapsulated proteins in the protein@Fe-fum were released by disintegrating the uncoated protein@Fe-fum. To this end, ethanolic suspension of protein@Fe-fum was centrifuged at 16 900 RCF for 5 min. The pellet was then redispersed in citrate buffer (10 mM) to the same volume as before to allow for the complete dissociation of protein@Fe-fum. Afterward, to quantify the protein loading in protein@Fe-fum, a BCA assay was performed according to the protocol provided by the manufacturer (Pierce BCA Protein Assay Kit, Thermo). The albumin standard (BSA), provided in the kit, was diluted sequentially to concentrations between 0 and 250 μg/mL in citrate buffer or fumaric acid to obtain the standard curve for quantifying the concentration of encapsulated protein and initial protein, respectively. The absorption at 562 nm was measured using a SpectraFluor Plus microplate reader S4 (Tecan, Grödig, Austria).

### Confocal Microscopy

The fluorescence microscope images were taken with a Zeiss Observer SD spinning disk confocal microscope using a Yokogawa CSU-X1 spinning disc unit and an oil objective with 63× or 100× magnification (1.40 N.A.) and BP 525/50 (green channel) and LP 690/50 filters (red channel). For excitation, a 488 nm and a 639 nm laser were used. The images were processed with the ImageJ v2.35 software.

### Cell Culture

HeLa cells (a human cervical carcinoma cell line) and HeLa GFPTub cells (Hela cells stably expressing eGFP-tubulin) were cultivated in Dulbecco’s modified Eagle’s medium (DMEM, Gibco) with 10% (v/v) fetal bovine serum (FBS, Gibco) and 1% (v/v) penicillin–streptomycin (Gibco). Cells were grown in a cell culture incubator (Heracell) at 37 °C with 5% carbon dioxide.

### Cell Viability Assay (MTT Assay)

The cytotoxicity of the lipid-coated, calcein-loaded BSA@Fe-fum was studied using an MTT assay (Thermo Fisher). HeLa cells were seeded onto 96-well plates with a density of 5000 cells per well 24 h before treatment. The cells were then treated with different concentrations of lipid-coated, calcein-loaded BSA@Fe-fum (0–240 μg/mL) and each concentration in triplicate. Cells without treatment were used as a control group. After 48 h, the MTT assay was performed. For this, cells were washed twice with PBS and then incubated for 2 h in DMEM containing 500 μg/mL MTT (3-(4,5-dimethylthiazol-2-yl)-2,5-diphenyltetrazolium bromide) in the incubator (37 °C, 5% carbon dioxide). Subsequently, the supernatant was removed, and the cells were lysed by incubating the plate at −80 °C for at least 20 min. Next, the resulting purple crystals were dissolved in 100 μL of DMSO per well and the absorption at 590 nm was measured using a SpectraFluor Plus microplate reader S4 (Tecan, Grödig, Austria). Cell viability was calculated as the ratio of the absorption of wells with treated cells relative to wells with untreated control cells.

### In Vitro Activity Assay of HRP

For the in vitro HRP enzymatic activity assay, the Fe-fum NPs and HRP@Fe-fum were centrifuged at 16 900 RCF for 5 min. Subsequently, the pellet was redispersed in citrate buffer to disintegrate the NPs and release the encapsulated HRP. Then, the decomposed NPs at a volume corresponding to 4 μg of HRP were added to 1.5 mL of an aqueous solution containing 2500 μM H_2_O_2_ and Amplex UltraRed (final concentration 50 μM). The enzyme activity was determined by monitoring the fluorescence of mixtures at an excitation/emission of 571/584 nm. Spectra were recorded with an MD-5020 setup from PTI Photon Technology International. HRP kinetics was performed using free HRP (at a concentration of 0.03 and 0.06 ng/mL) in presence of degraded Fe-fum NPs or degraded HRP@Fe-fum (at a concentration of 30 and 60 ng/mL) and 2500 μM H_2_O_2_ and Amplex UltraRed concentrations between 0 and 100 μM. The resulting fluorescence intensity was measured at different time points with a SpectraFluor Plus microplate reader S4 (Tecan, Grödig, Austria) and 535/590 excitation/emission filters. *K*_M_ and *k*_cat_ were determined via a fit to v_0_/E_0_ vs. Amplex UltraRed concentration according to the Michaelis–Menten equation as described in the literature.^34^

### Inducing Protein Release in the Cytosol (Glucose Shock)

To induce protein release from the endosome into the cytosol, a glucose shock was applied. For this purpose, the cell medium was removed, and cells were exposed to a 1 M solution of glucose in DMEM for 6 min. The cells were then washed twice with PBS to remove the glucose completely before fresh DMEM or FluoroBrite DMEM (in case of imaging) was added to the cells.

### Intracellular Activity of HRP

HeLa cells were seeded on ibidi 8-well plates with a density of 5000 cells per well. The next day, cells were treated with lipid-coated, calcein-loaded HRP@Fe-fum and lipid-coated, calcein-loaded Fe-fum NPs as the negative control. After 3 days, the supernatant was removed, and a glucose shock was applied as described above. After 6 min, the cells were incubated with 200 μL of DMEM containing 2500 μM H_2_O_2_ and 50 μM Amplex UltraRed for 30 min at room temperature. Then, the cells were washed with PBS and imaged.

### Degradation Studies

To study degradation of the Fe-fum NPs, 90 μg of uncoated HRP@Fe-fum with Atto633-labeled HRP were centrifuged and dissolved in either water at the indicated pH, 10 mM citrate buffer, or cysteine at the indicated concentration in water. If not stated otherwise, the NPs were centrifuged after 7 min and the absorption spectrum of the supernatant was measured using a Thermo Scientific NanoDrop 2000c spectrometer. For analysis, the absorption at 633 nm was used. For kinetics studies, 1 mL of a 20 mM cysteine solution was used to dissolve 900 μg of HRP@Fe-fum. After the indicated time intervals, 100 μL of samples were taken and centrifuged and the supernatant analyzed with respect to the absorption at 633 nm using a Thermo Scientific NanoDrop 2000c spectrometer.

### Compatibility of the Synthesis Process of NPs with RNP (In Vitro Cleavage Assay)

To confirm the compatibility of the fumaric acid (at different pH) as well as the Fe-fum NP synthesis with the RNP, an in vitro cleavage assay was applied. Briefly, a linearized plasmid containing the EGFP gene (linear pEGFPLuc) was incubated with the RNP and the activity of the RNP was visualized via the existence of bands resulting from cleaved plasmid in a 2% agarose gel. To study the effect of fumaric acid on the activity of RNP, initially the RNP was incubated with fumaric acid for 10 min. Then, the pEGFPLuc was treated with RNPs in 1× Cas9 nuclease reaction buffer (New England Biolabs, NEB) for 1 h. The activity of the RNPs was compared via the amount of cleaved pEGFPLuc in the agarose gel.

To investigate the compatibility of the Fe-fum NP synthesis with RNPs, the RNP@Fe-fum were first decomposed by redispersing the NP pellet in cysteine (80 mM, pH 5) for 10 min. Then, the pEGFPLuc was treated with the decomposed Fe-fum NPs containing the released RNP. Next, samples were incubated with EDTA 5 mM for 10 min to chelate the free iron ions, which interfere with electrophoresis. Then, activity was measured using electrophoresis as described above.

### RNP Genome-Editing Efficiency Study (Cellular Knockout Experiments)

HeLa GFPTub cells (GFP-expressing HeLa cells) were used as an RNP-transfection cell model. Twenty-four hours before treatment, cells were seeded at a density of 5000 cells per well onto 96-well plates in 100 μL of DMEM. The next day, cells were treated with lipid-coated, calcein-loaded RNP@Fe-fum containing 75 or 110 nM Cas9. Cells treated with corresponding concentrations of Fe-fum NPs without RNPs, with a control RNP that does not target a genome sequence, as well as cells treated only with HEPES buffer served as negative controls. As the positive control, the RNP complex formulated with Lipofectamine CRISPRMAX was applied. All treatments were performed in triplicate. After 48 h, glucose shock was performed in case of the cells treated with Fe-fum NPs, as described above. Then, every 48 h, the cells were trypsinized and passaged into a new plate. After two passages, the fluorescence signal resulting from the coloaded calcein disappeared allowing for analysis of GFP expression without artifacts. Forty-eight hours after the second passaging, cells were harvested, and the knockout efficiency was determined by flow cytometry (CytoFLEX S, Beckman Coulter) as the percentage of GFP-negative cells after subtraction of unspecific GFP-negative population in HEPES-treated cells (gating strategy, see Figure S11). The GFP knockout was visualized by imaging using an ImageXpress Micro XLS (Molecular Devices) with a 40× objective and a GFP filter. The resulting images were evaluated with the ImageJ v2.35 software.

### Protective Feature of NPs against the Acidic Condition

To study the capability of Fe-fum NPs to protect RNPs from acidic conditions, the encapsulated RNPs in the lipid-coated, calcein-loaded RNP@Fe-fum, as well as free RNP (both containing 75 nM Cas9), were exposed to a low pH environment by addition of HCl (final pH was 3.5). After a 10 min or overnight incubation, the pH of the environment was neutralized with NaOH. Then, the activity of acid-treated free RNP (transfected with Lipofectamine CRISPRMAX) and encapsulated RNP, as well as a nontreated group of samples, was compared by performing a cellular knockout efficiency experiment as described above.

### Stability of the Encapsulated RNP Over Time

Lipid-coated, calcein-loaded RNP@Fe-fum and free RNP (both containing 110 nM Cas9) were stored at 4 °C for 2 months. The intracellular activity of old free RNP transfected with Lipofectamine CRISPRMAX and encapsulated RNP was compared with fresh samples by performing a cellular knockout efficiency experiment as described above.

## Supporting Information Available

Supporting Figures S1–S13 containing SEM size analysis, XRD, IR data, GFP fluorescence analysis, additional cleavage assays, nanoparticle degradation, schematic of knockout experiment, additional knockout results from FACS and microscopy, and FACS gating strategy (PDF)
