# Supplementary material for: Biomimetic Mineralization of Iron-Fumarate Nanoparticles for Protective Encapsulation and Intracellular Delivery of Proteins
Source: Chem Mater. 2022 Oct 3;34(19):8684–93. doi: 10.1021/acs.chemmater.2c01736 (PMC9558304; doi:10.1021/acs.chemmater.2c01736)
Supplement: Supplementary file 1 — cm2c01736_si_001.pdf [file cm2c01736_si_001.pdf]

Supporting Information

Biomimetic mineralization of iron-fumarate nanoparticles for protective encapsulation and intracellular delivery of proteins

Negar Mirzazadeh Dizaji[1], Yi Lin[1], Thomas Bein[1,2], Ernst Wagner[1,2], Stefan Wuttke[2,3,4], Ulrich Lächelt*[1,2,5], Hanna Engelke*[2,6]

[1]Faculty for Chemistry and Pharmacy, Ludwig-Maximilians-Universität München, Butenandtstr. 5-13, 81377 Munich, Germany

[2]Center for NanoScience, Ludwig-Maximilians-Universität München, Schellingstr. 4, 80799 Munich, Germany

[3]Basque Center for Materials (BCMaterials), UPV/EHU Science Park, 48940 Leioa, Spain.

[4] Ikerbasque, Basque Foundation for Science, 48009 Bilbao, Spain.

[5]Department of Pharmaceutical Sciences, University of Vienna, Josef-Holaubek-Platz 2, 1090 Vienna, Austria

[6]Department of Pharmaceutical Chemistry, Institute of Pharmaceutical Sciences, University of Graz, Humboldtstr. 46, 8010 Graz, Austria

*Correspondence to: ulrich.laechelt@univie.ac.at (UL) and hanna.engelke@uni-graz.at (HE)

Supplementary Figures:

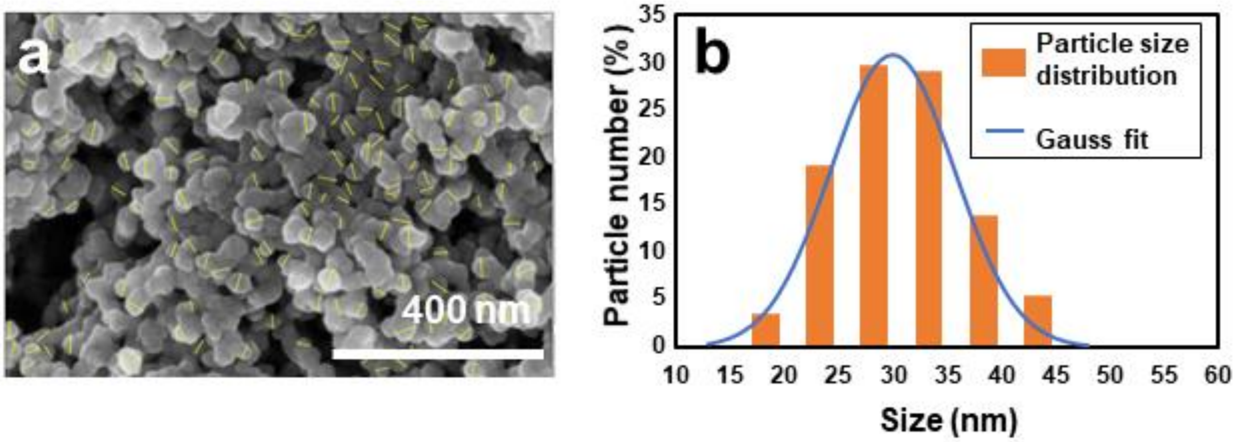

Figure S1. SEM micrograph of Fe-fum NPs **(a)** and size distribution of Fe-fum NPs obtained from SEM micrographs **(b)**. The particle size distribution was determined by manually measuring the diameter of 152 particles (a, yellow lines). This results in an average particle diameter of ~ 30 nm with a standard deviation of 5.7 nm.

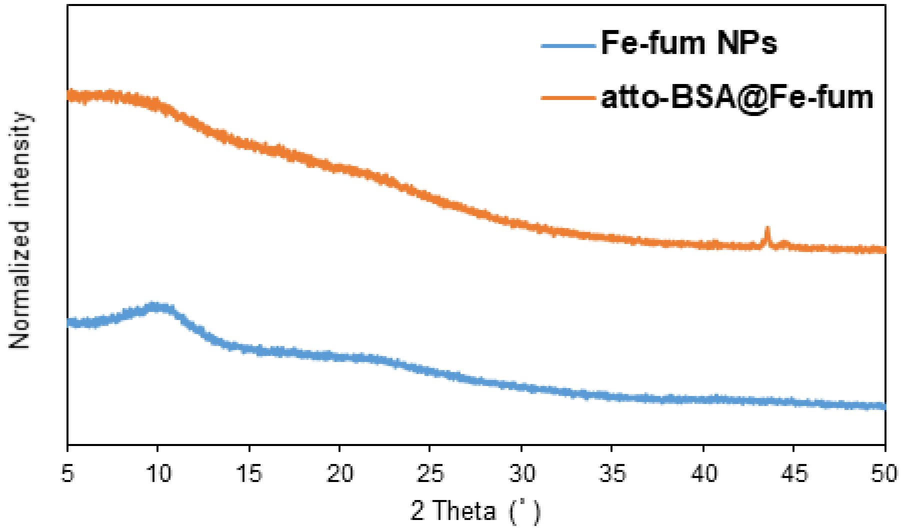

Figure S2. XRD measurements of Fe-fum NPs (blue) and fluorescently labelled BSA biomimetically mineralized into Fe-fum NPs (atto-BSA@Fe-fum; orange).

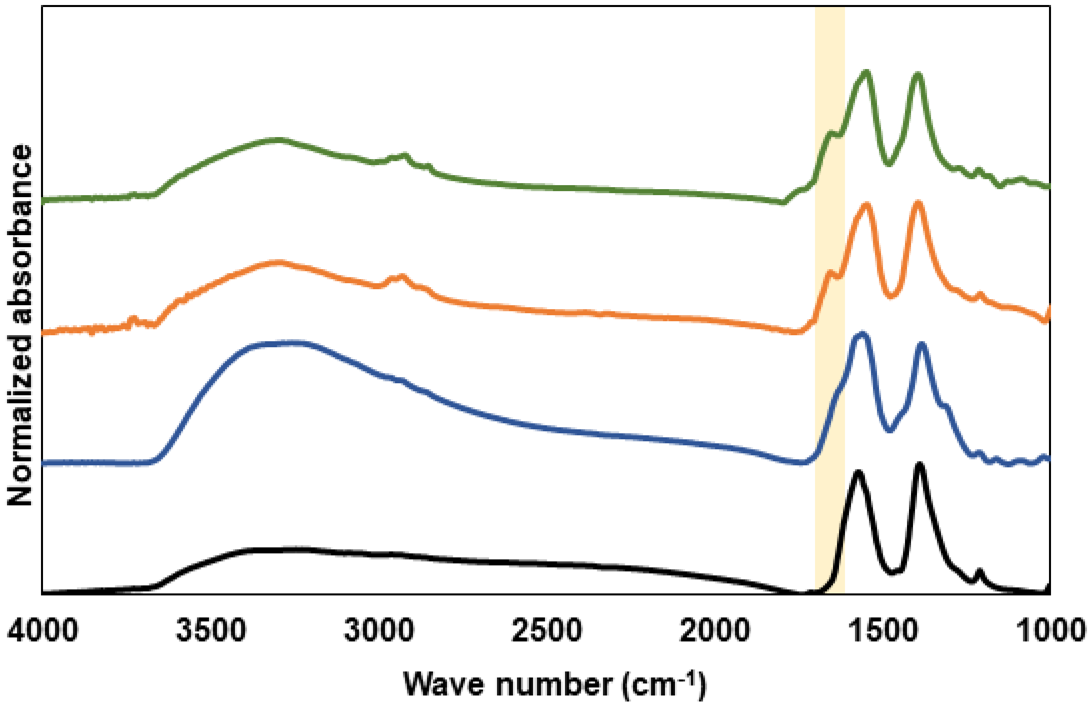

Figure S3. IR spectra of fumaric acid (black), Fe-fum NPs (blue), HRP@Fe-fum (orange) and RNP@Fe-fum (green). Appearance of a peak (marked in yellow) in the range of 1640-1660 cm^{-1} (corresponding to the amide I band, mainly from C=O stretching vibrations) in the spectra of HRP@Fe-fum and RNP@Fe-fum confirms the successful incorporation of HRP and RNP in the Fe-fum NPs.

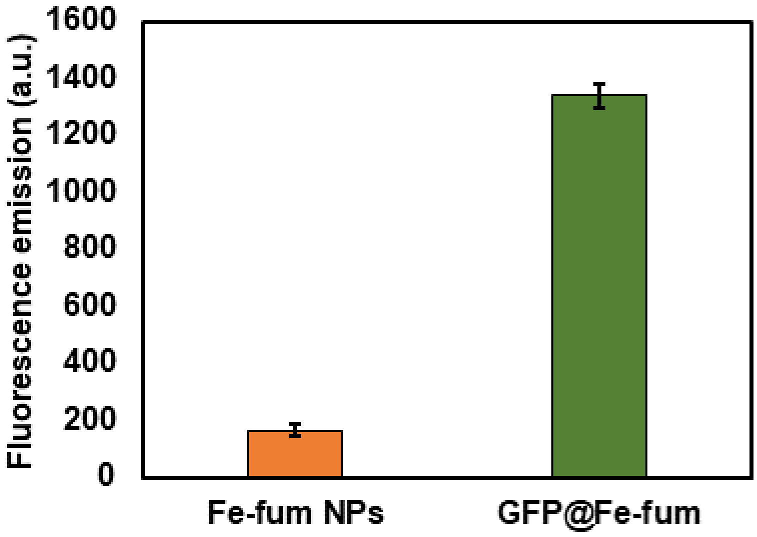

Figure S4. GFP fluorescence emission of degraded Fe-fum NPs synthesized with (green) and without (orange) GFP. GFP fluorescence is preserved during the NP synthesis and ethanol washing steps.

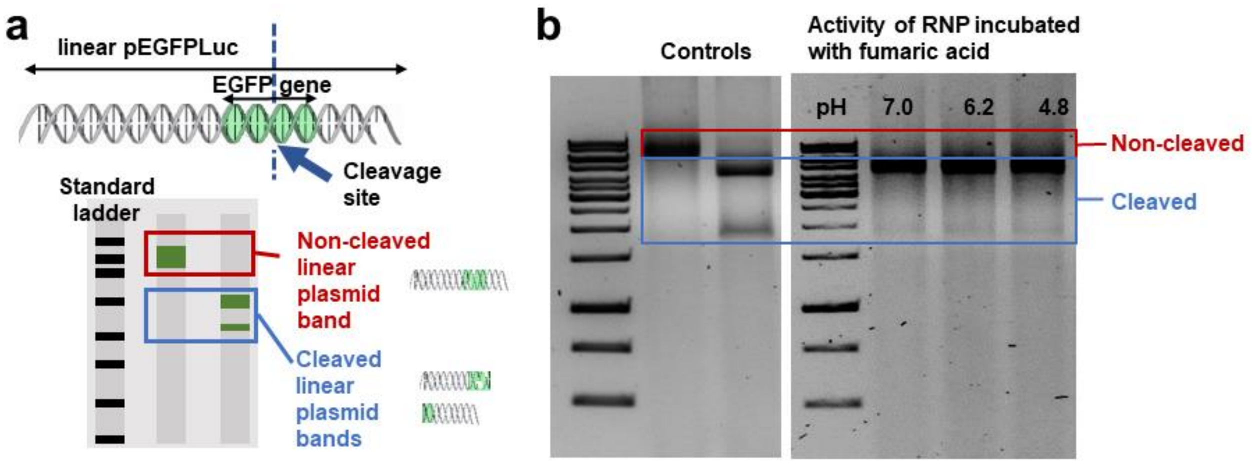

Figure S5. a) Schematic of the *in vitro* cleavage assay and **b)** the bands of the pEGFPLuc plasmid and fragments resulting from cleavage by RNP in an agarose gel.

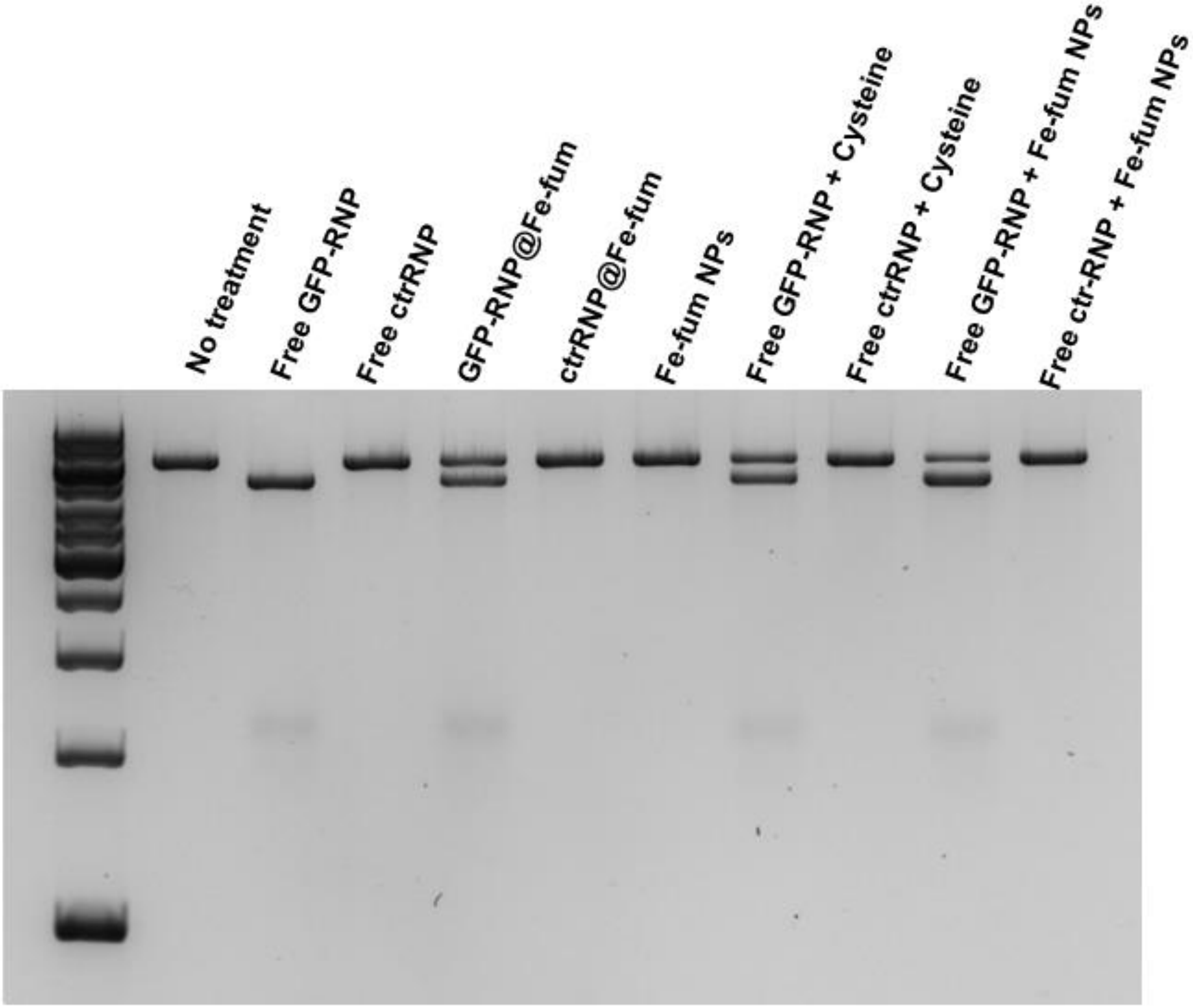

Figure S6. In vitro cleavage assay confirming RNP-activity after Fe-fum NP synthesis. The linear plasmid was treated with free RNP as positive control and with RNP@Fe-fum and Fe-fum NPs both decomposed by a 10 min incubation with cysteine. The effect of cysteine and Fe-fum NPs' ingredients on the activity of RNP was studied by incubation of free RNP with cysteine and decomposed NPs before performing the cleavage assay. As an additional negative control, RNP with control RNA without target sequence in the genome (ctrRNP) was used for each RNP treatment.

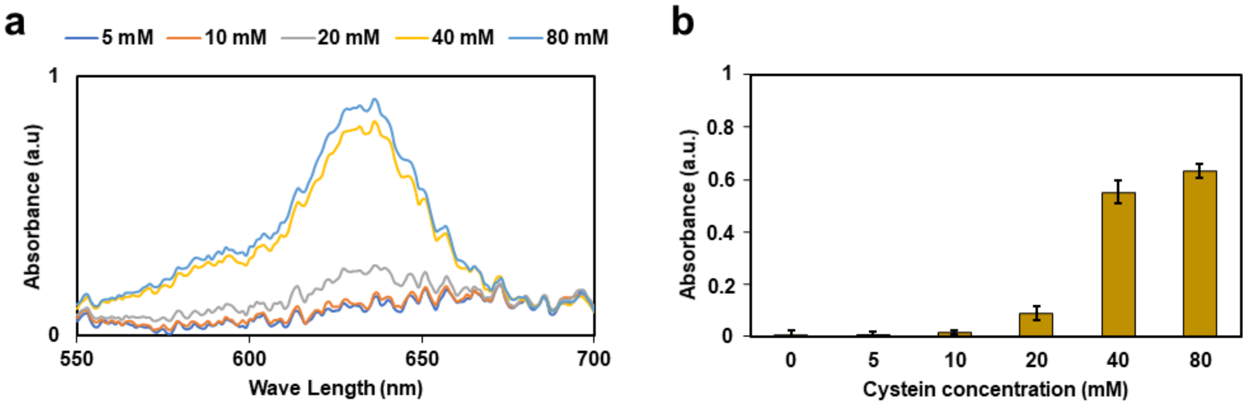

Figure S7. Concentration dependent degradation of Atto633-labeled-HRP@Fe-fum. (a) Absorbance spectra of Atto633-labeled-HRP@Fe-fum (supernatant) after treatment with increasing concentrations of cysteine, as indicated by the color code. The absorbance of the supernatant at 633 nm is increasing due to the Atto633-labeled HRP released from the increasingly degraded nanoparticles (b) Quantification of the absorbance at 633 nm confirms the increasing degradation with increasing concentration of cysteine.

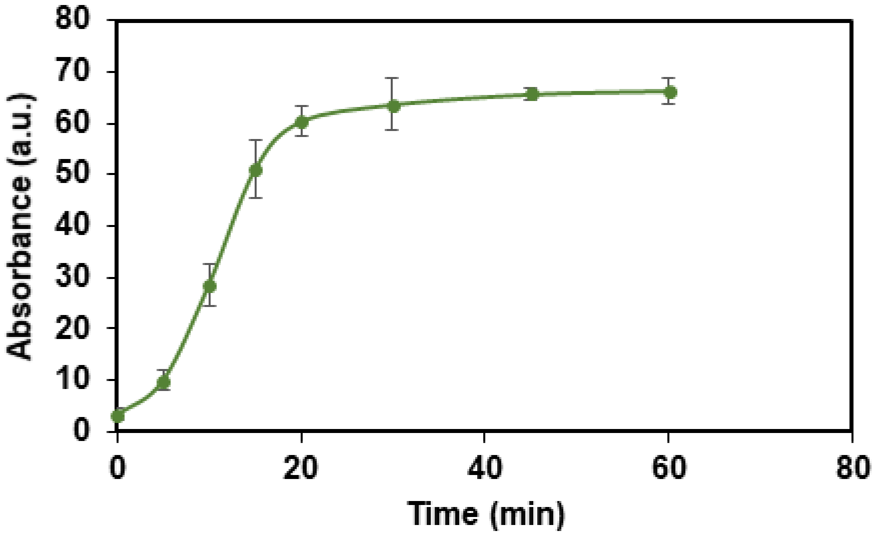

Figure S8. Degradation kinetics of Atto633-labeled HRP@Fe-fum in 20 mM cysteine.

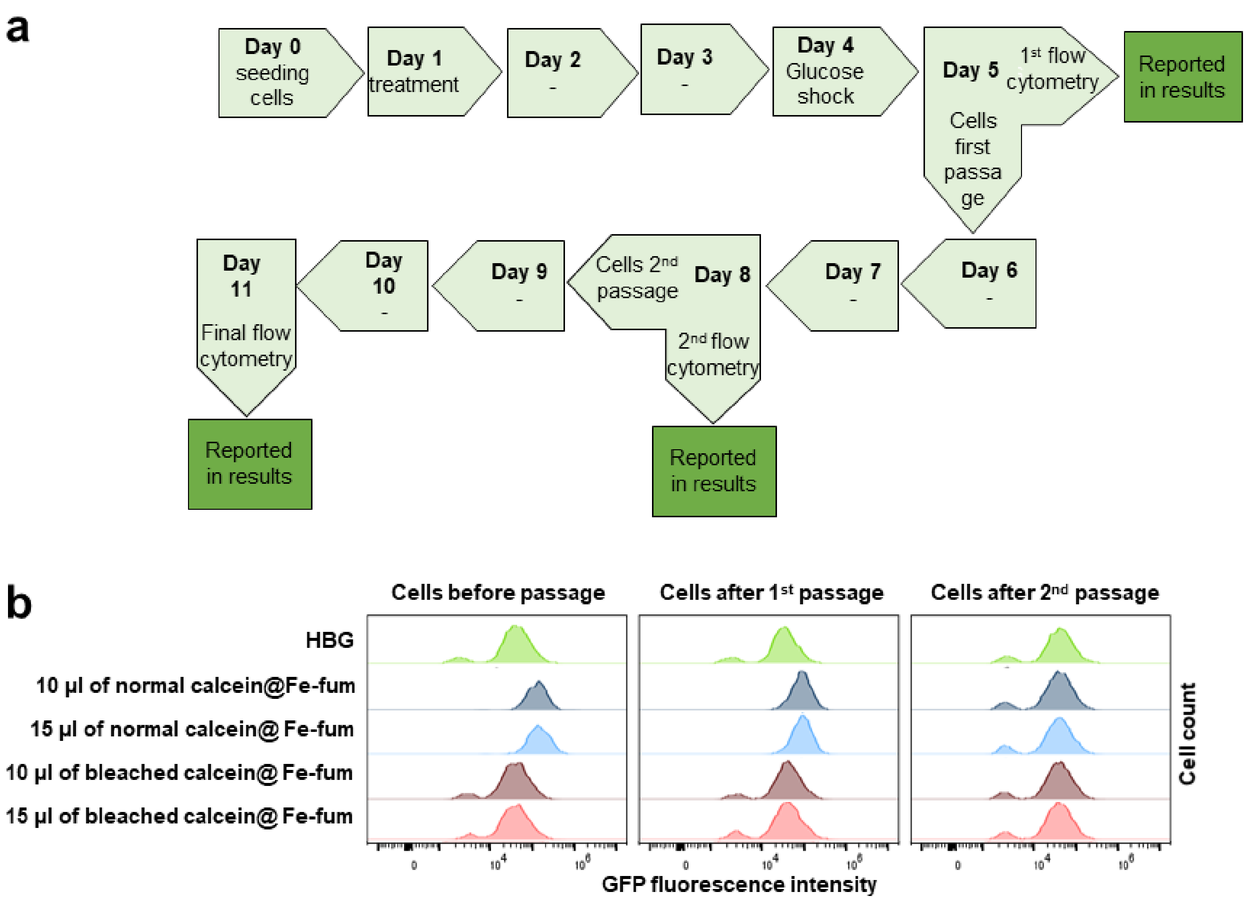

Figure S9. a) Schematic of the steps applied for studying the knockout efficiencies as well as the exclusion of interference with calcein in the GFP channel. **b)** Flow cytometry analysis of GFP fluorescence and calcein interference in HeLa cells after sequential splitting of the cells. Stably GFP expressing HeLa cells (HeLa GFPTub cells) were incubated with lipid-coated Fe-fum NPs loaded with (normal or bleached) calcein (calein@Fe-fum) and the GFP fluorescence of cells was measured after 5 days (before passage), 8 days (after first passage) and 11 days (after second passage). Cells incubated with HBG buffer only (HBG) served as control. Flow cytometry results confirm that the calcein interference with the GFP signal is negligible after the second splitting.

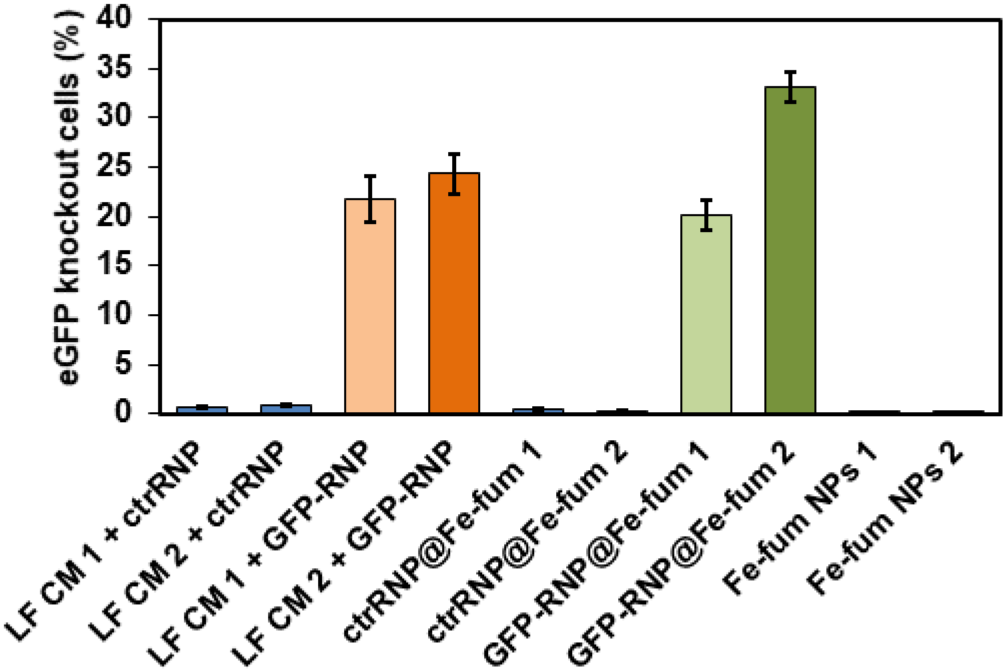

Figure S10. One additional experiment on the knockout efficiency of lipid-coated, calcein-loaded RNP@Fe-fum or Lipofectamine CRISPRMAX (LF CM) containing a control guide RNA without target in the genome (ctrRNP) or GFP-specific guide RNA (GFP-RNP). LF CM1 and Fe-fum 1 correspond to a concentration of 75 nM RNP, LF CM2 and Fe-fum 2 to a concentration of 110 nM RNP. The experiment confirms the specificity of the GFP knockout.

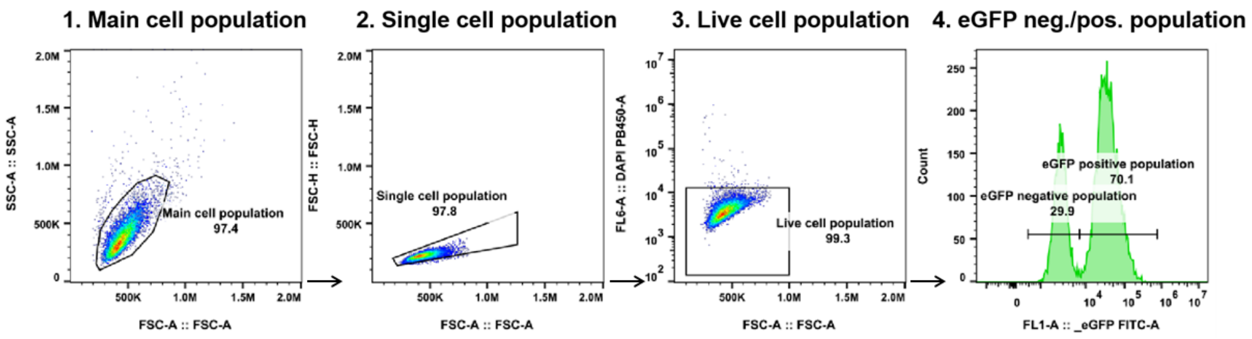

Figure S11. FACS gating strategy for experiments shown in Fig. 5 and Fig. S10. Briefly, 1) Forward versus side scatter (FSC-A vs SSC-A) gating is used to identify cell population of interest and exclude cell debris. (2) A forward scatter height (FSC-H) vs. forward scatter area (FSC-A) density plot is used to exclude doublet cells. (3) A forward scatter area (FSC-A) vs. DAPI area (DAPI-A) density plot is used to identify live cells. (4) eGFP single parameter histogram is used for identifying eGFP negative and positive cell population.

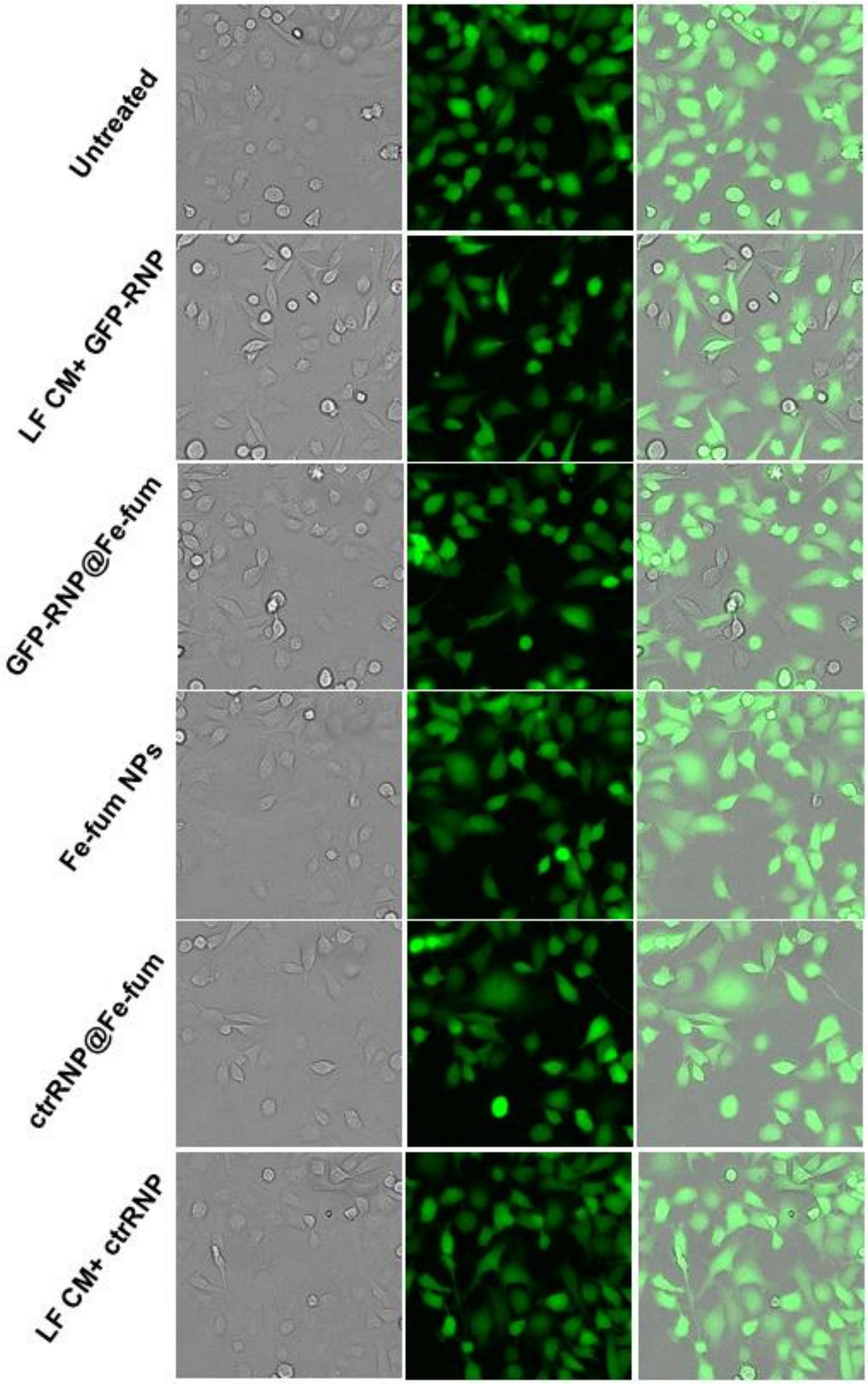

Figure S12. Fluorescence and brightfield high-content microscopy of HeLa GFPTub cells after treatment with GFP-RNP@Fe-fum, LF CM+GFP-RNP or controls. The experiment confirms the specific GFP knockout as determined by FACS in Fig. S10. Scale bar: 100 µm.

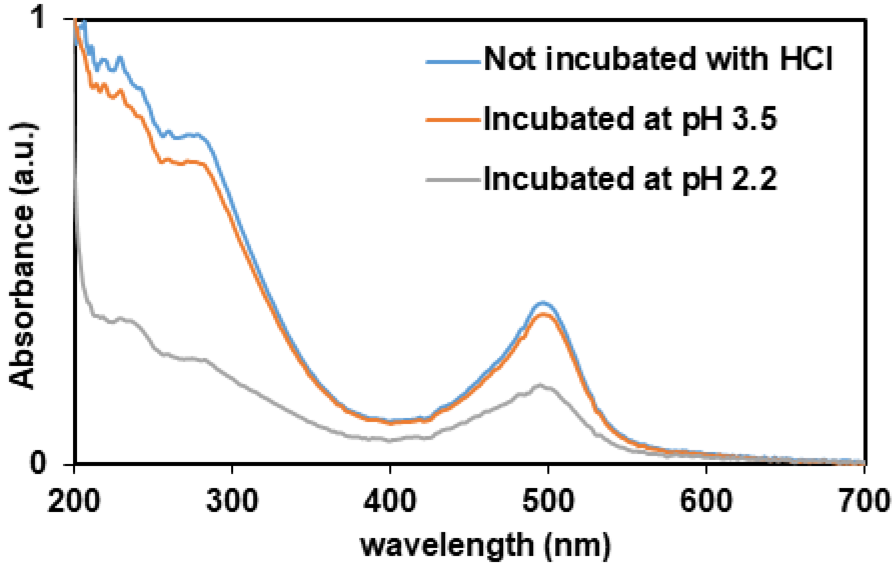

Figure S13. Absorbance of lipid-coated Fe-fum NPs after incubation at pH 2.2 and 3.5 overnight. The similarity of the spectra of Fe-fum NPs incubated at pH 3.5 and of Fe-fum NPs that were not incubated with HCl confirms the stability of Fe-fum NPs in acidic condition down to pH 3.5. The intensity changes in the spectrum of Fe-fum NPs incubated at pH 2.2 suggest their partial degradation at this low pH.

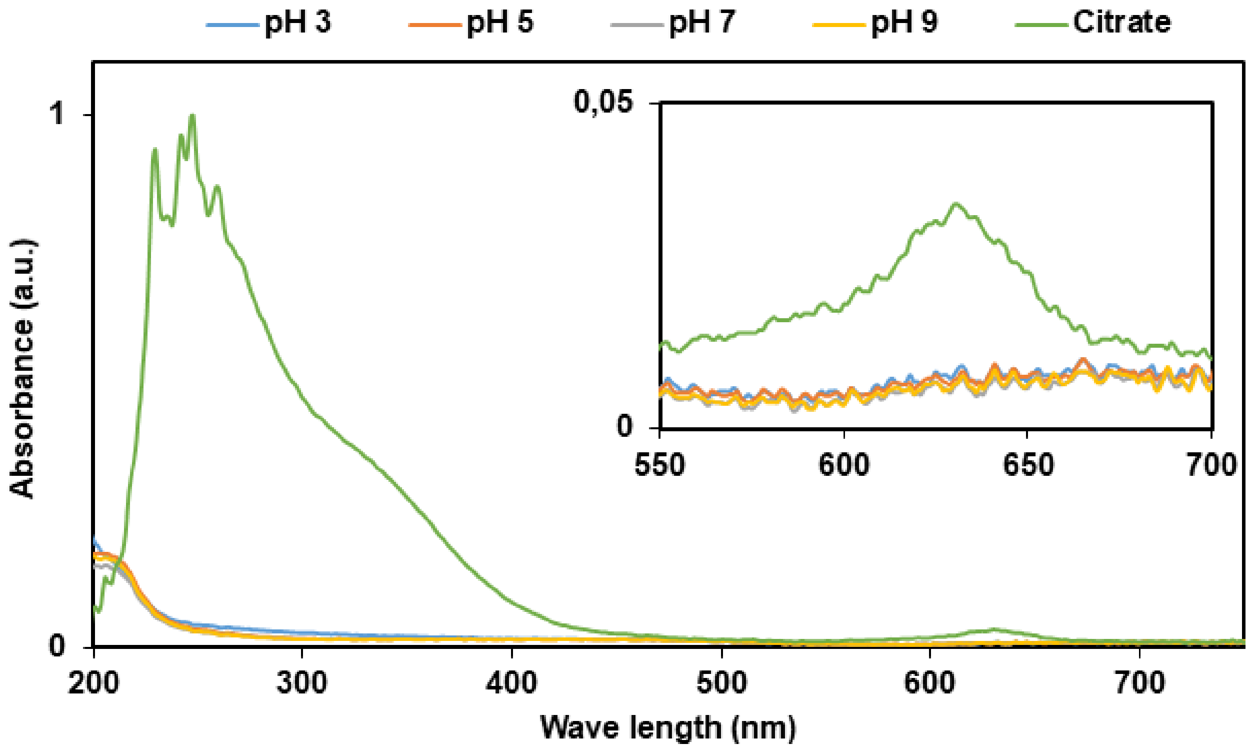

Figure S14. Absorbance of the supernatant of Atto633-HRP@Fe-fum NPs after incubation at the indicated pH values, as well as in citrate solution. No increase in absorbance at 633 nm is detected after incubation within the range between pH 3-9 indicating that the NPs do not degrade and release Atto633-labeled HRP into the supernatant. In the case of citrate, NPs are degraded and release Atto633-labeled HRP into the supernatant leading to an increase in absorbance at 633 nm.
